# On-Surface Synthesis of Ni-Porphyrin-Doped Graphene Nanoribbons

**DOI:** 10.1021/acsnano.4c09188

**Published:** 2024-11-25

**Authors:** Matthew Edmondson, Michael Clarke, James N. O’Shea, Qiang Chen, Harry L. Anderson, Alex Saywell

**Affiliations:** † School of Physics and Astronomy, University of Nottingham, Nottingham NG7 2RD, U.K.; ‡ Department of Chemistry, Chemistry Research Laboratory, University of Oxford, Oxford OX1 3TA, U.K.

**Keywords:** graphene nanoribbons, on-surface synthesis, porphryin, polymer, scanning probe microscopy (SPM), electrospray deposition, photoelectron spectroscopy

## Abstract

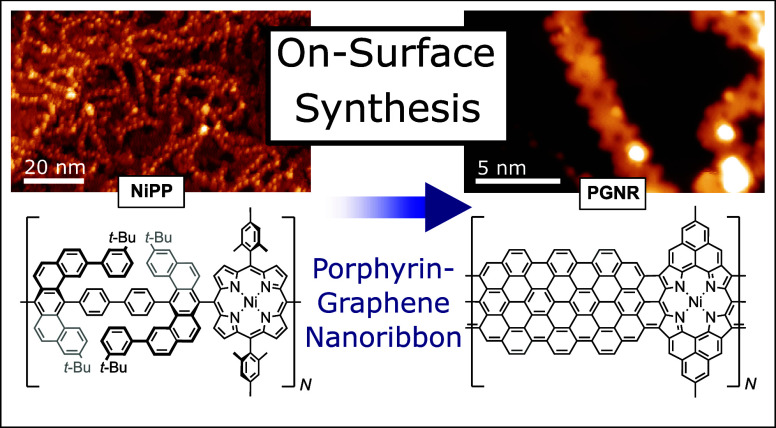

On-surface synthesis of functional molecular structures provides a route to the fabrication of materials tailored to exhibit bespoke catalytic, (opto)electronic, and magnetic properties. The fabrication of graphene nanoribbons via on-surface synthesis, where reactive precursor molecules are combined to form extended polymeric structures, provides quasi-1D graphitic wires that can be doped by tuning the properties/composition of the precursor molecules. Here, we combine the atomic precision of solution-phase synthetic chemistry with on-surface protocols to enable reaction steps that cannot yet be achieved in solution. Our focus of this work is the inclusion of porphyrin species within graphene nanoribbons to create porphyrin-fused graphene nanoribbons. A combination of scanning tunneling microscopy and photoelectron spectroscopy techniques is used to characterize a porphyrin-fused graphene nanoribbon formed on-surface from a linear polymer consisting of regularly spaced Ni-porphyrin units linked by sections of aryl rings which fuse together during the reaction to form graphitic regions between neighboring Ni-porphyrin units.

## Introduction

Graphene nanoribbons (GNRs) possess fascinating electronic properties with the potential for applications within (opto)electronic devices.^1^ Studies of GNRs often focus upon the on-surface synthesis of extended graphitic polymers formed from the Ullmann-type coupling of halogen functionalized precursors, such as bianthracene units.^2^ In common with many on-surface synthesis protocols, the initial, intermediate, and product states can be studied by scanning tunneling microscopy (STM) and atomic force microscopy (AFM) to provide submolecular insights into reaction pathways and resultant structures.^3^ Recently, the on-surface synthesis of open-shell nanographenes which exhibit π-magnetism^4^ has led to contemplation on their use within spintronic devices and quantum computing architectures.^5^ There is also significant interest in achieving doped and heterostructured GNRs, with the aim of allowing tunable bandgap and Fermi-level engineering. The chemical modification of the reactive precursor molecules, which are the building blocks of GNRs, is a widely explored route to providing functional groups at the periphery^6,7^ and within the core of the GNR; boron^8^ and nitrogen^9−11^ are frequently used as dopant species.

While an on-surface synthesis approach to the formation of GNRs has allowed polymers with a range of structures to be fabricated, there are still significant challenges relating to the selectivity and efficiency of such on-surface reactions. Our proposed methodology utilizes the atomic precision of solution-phase chemistry to form highly regular polymeric precursor species and to employ on-surface protocols to enable reaction steps which are not facile in solution (e.g., the dehydrogenative cyclization reaction step required to produce conjugated graphitic materials). Our focus is on the inclusion of porphyrin species within the graphene nanoribbons, giving rise to porphyrin-fused graphene nanoribbons (PGNRs). Porphyrins are robust molecular species, amenable to functionalization by pendant chemical groups at the periphery and metalation of the macrocyclic core. Porphyrin species have been well-studied by a number of surface science approaches including photoelectron spectroscopies (PES)^12^ and scanning probe microscopies (including STM and AFM).^13^ The functionalization of graphene structures has been demonstrated by fusion of tetrapyrroles (free-base porphyrins, 2H-P) to the edges of extended graphene structures^14^ and by the on-surface synthesis of GNRs with porphyrin units fused at regular distances along the edge of the nanoribbon,^15^ while the inclusion of porphyrin groups within GNRs has been achieved by on-surface methods but frequently results in short oligomers,^16,17^ exhibiting a high defect density.^18^ Importantly, the inclusion of metal functionalized porphyrins provides a scheme for doping these graphitic species,^17^ allowing access to spin states^18,19^ and for the incorporation of the catalytic properties of such species.^13^

**Figure 1:**
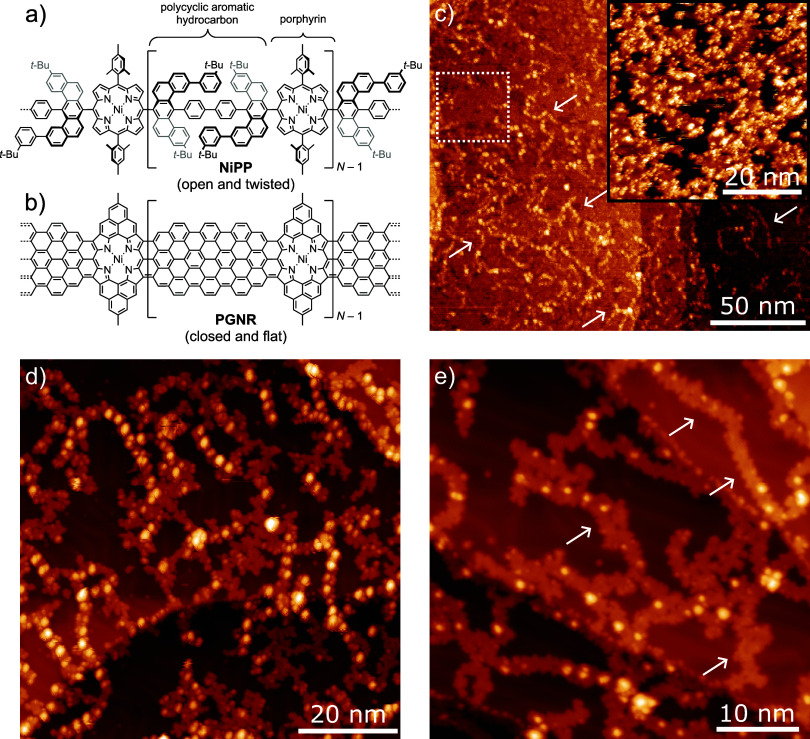
Overview of the on-surface synthesis of a porphyrin-fused graphene nanoribbon (**PGNR**). (a) Chemical structure of **NiPP**. (b) Proposed structure of the reaction product, **PGNR**, based upon known on-surface cyclization steps^16,20−23^ (degree of polymerization is expected to be up to *N* = 54). STM images of the as-deposited **NiPP** on Au(111) (c) as deposited, (d) following annealing to 350 °C, and (e) following annealing to 450 °C. Image parameters: (c) −1.5 V, 50 pA, *T* = 78 K (inset −2.0 V, 50 pA, *T* = 78 K), (d) −2.0 V, 50 pA, *T* = 4.7 K, and (e) −2.0 V, 20 pA, *T* = 4.7 K.

A challenge for a combined solution-phase and on-surface approach is the transfer of large (over 100 nm in length in the case of the polymers used in this work^24^), thermally labile polymers from solution to the ultrahigh vacuum (UHV) environments required for precision PES and STM measurements. Over the last decades, electrospray-based deposition (ESD) procedures have been used to deposit a range of fragile, functional, and polymeric species onto substrates held under UHV conditions;^25−35^ including graphene nanoribbons.^36^ This has provided a route to the characterization of molecular structure and properties, by combining the spatial resolution of STM and AFM with the chemical and structural sensitivity of PES techniques (e.g., X-ray photoelectron spectroscopy (XPS), near-edge X-ray adsorption fine-structure (NEXAFS), and X-ray standing wave (XSW) techniques).^24,37−39^ In this work, we employ a combination of ESD, STM, XPS, NEXAFS, and XSW to characterize a porphyrin-fused graphene nanoribbon (**PGNR)** formed from the on-surface synthesis of a linear polymer consisting of regularly spaced Ni-porphyrin units linked by sections of aryl rings (**NiPP**—a nickel(II) porphyrin polymer, synthesized and characterized as reported previously^24^). **NiPP** is designed to fuse together to form graphitic regions between neighboring Ni-porphyrin units (see Figure 1a,b for proposed reaction scheme).

## Results and Discussion

Following ESD of **NiPP** on to a Au(111) substrate, STM characterization reveals the presence of long chain-like structures (see Figure 1c—several chains indicated by arrows). The chains exhibit significant flexibility and are observed to run continuously across step-edges and over other chains, which are similar to the appearance of previously characterized porphyrin-based polymers.^40^ The average degree of polymerization for the deposited material is expected to be about *N̅* = 34 (based upon GPC and MS analysis for a related polymer), which is equivalent to a polymer length of ∼85 nm (DFT calculations indicate that the Ni–Ni separation of neighboring porphyrins is ∼2.5 nm).^24^ The deposited chains are observed as continuous polymers that frequently cross; continuous chain lengths of up to 55 nm were observed within the acquired STM data. Between the chains are domains of material that cover the Au(111) surface (highlighted by white-dashed box and inset in Figure 1c). We assign the material between the chains to a codeposited contaminant (tentatively attributed to polydimethylsiloxane, PDMS, due to traces of silicon-grease, although the presence of solvent species, methanol and toluene, is not excluded; the presence of oxygen and silicon species is indicated by XPS analysis of samples prepared by this ESD methodology). The contaminant is removed after annealing at 350 °C (see Figure 1d), where clean areas on the Au(111) terraces can now be resolved along with the characteristic herringbone reconstruction^41^ (see SI). Along the chains, a periodic sequence of bright features is visible, which we assign to the alternating regions of the *polycyclic aromatic hydrocarbon* and *porpyhrin* sections of the polymer chain (see Figure 1a,d).

To initiate the on-surface ring-closing reaction that facilitates the formation of **PGNR**, the sample is annealed at 450 °C (see Figure 1e). Based on previous studies of on-surface ring-closing reactions,^16,20−23^ dehydrocyclization of **NiPP** is expected to occur at this temperature. It is clear that regions of the chains now have a reduced corrugation (i.e., “flatter” appearance in STM topographs; see regions highlighted by arrows in Figure 1e). However, STM characterization of the surface reveals bright protrusions along the chains, which we assign to the presence of non ring-closed material^22^ and tertiary butyl (*tBu*) groups.

**Figure 2:**
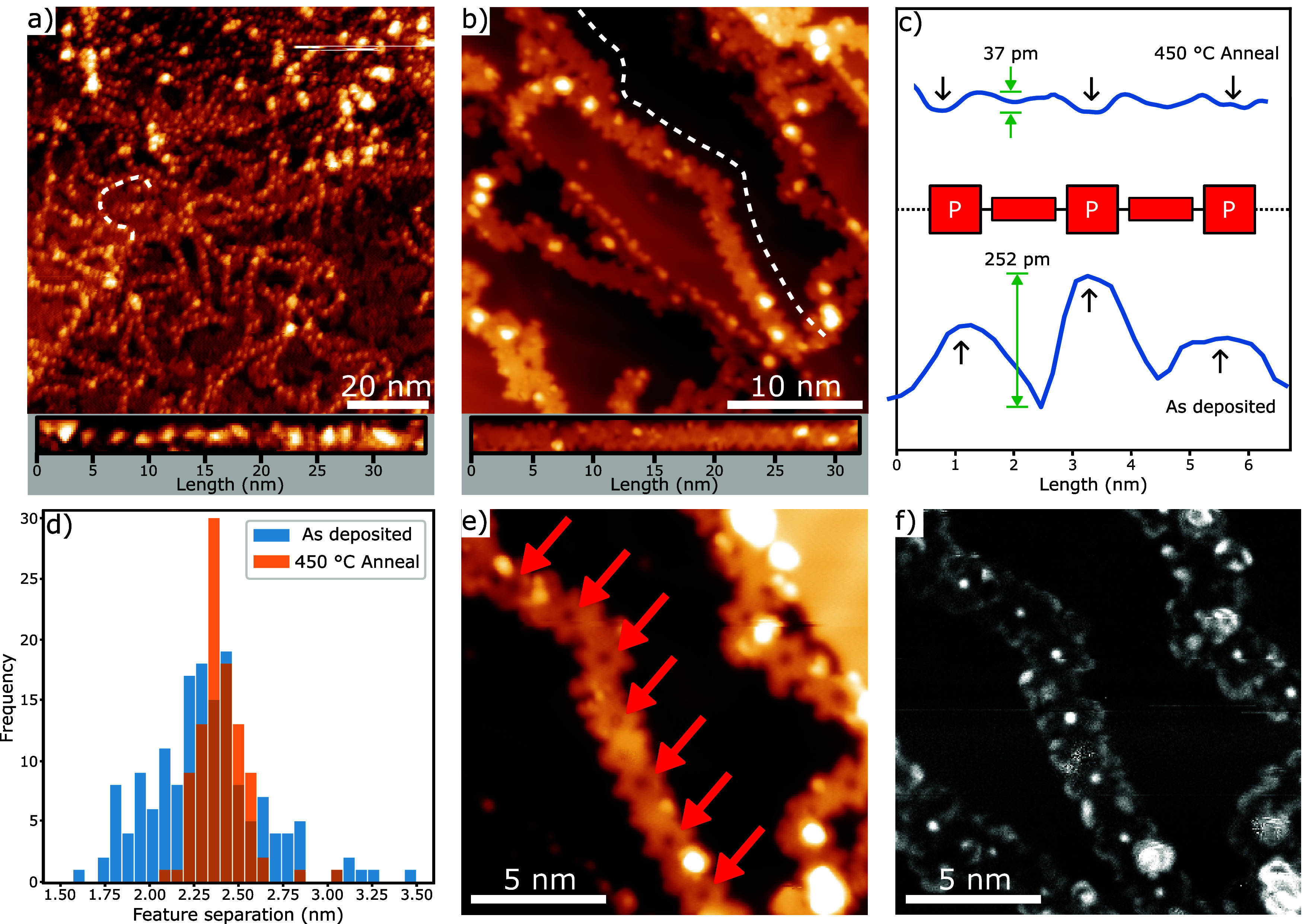
Details of periodicity and on-surface synthesis of **PGNR**. STM topographs of (a) **NiPP** following annealing to 250 °C and (b) **PGNR** formed by on-surface synthesis following annealing to 450 °C [panels below STM data show topography along the line profiles for the chains indicated within the image]. (c) Line profiles along the **NiPP** and **PGNR** chains; line profiles presented are segments containing two repeat units, acquired along the chains indicated in STM images (a) and (b). (d) Histogram showing the separation between periodic features along **NiPP** and **PGNR**. (e) STM topograph showing a close-up of a **PGNR** section; red arrows indicate features assigned to the center of the porphyrin macrocycles. (f) *dI*/*dV* map of the **PGNR** chain in (e); under these conditions, the Ni atoms at the core of the porphyrin species appear as bright features (assigned to the HOMO of the Ni-porphyrin subunit). STM image parameters: (a) −2.0 V and 50 pA, (b) −2.0 V, 100 pA, (e,f) −1.9 V, and *V*_Osc_ = 20 mV, 100 pA. All images acquired at 4.7 K.

Additional details about the chains can be obtained by measuring the periodicity along the length of the polymer chains. Figure 2a,b shows overview STM images of **NiPP** on Au(111) following annealing to 250 and 450 °C, respectively. The corrugation along the chain can be visualized as a line profile (the path that the STM tip would follow along the polymer chain) and is shown in Figure 2c for **NiPP** and **PGNR** materials. The periodicity of the chains is measured to be 2.32 nm, for the as-deposited material, and 2.40 nm postanneal (see Figure 2d), which is in excellent agreement with the expected periodicity of **NiPP** (∼2.5 nm^24^). Additionally, close-up STM topographs (Figure 2e) reveal the position of the periodic depressions to be at the center of the porphyrin macrocycle, which is indicated by red arrows. While the topographs show the cores to appear as depressions, differential conductance maps (dI/dV maps) acquired over the range of +2 to −2 V reveal that at negative sample biases in the range of −2 to −1.2 V, the cores appear bright (Figure 2f) which we assign to a contribution from a highest occupied molecular orbital-derived (HOMO-derived) valence band with a significant contribution from the Ni-porphyrin subunit of the nanoribbon (we note that dI/dV spectroscopy measurements of Ni-porphyrin species^42^ exhibit a resonant feature over a similar energy range, suggesting that the molecular HOMO may contribute to the HOMO-derived valence band for the **PGNR**). It is clear that the **PGNR** material exists as short sections of linear chains interspersed by “kinks” which result in a small change in chain direction. We assign such kinks to alternative carbon topologies formed during the cyclization step of the reaction (the formation of 5- or 7-membered rings, as opposed to 6-membered rings, would result in a deviation from linearity along the chain). However, it is possible that such kinks arise from defects present within the as-deposited material.

**Figure 3:**
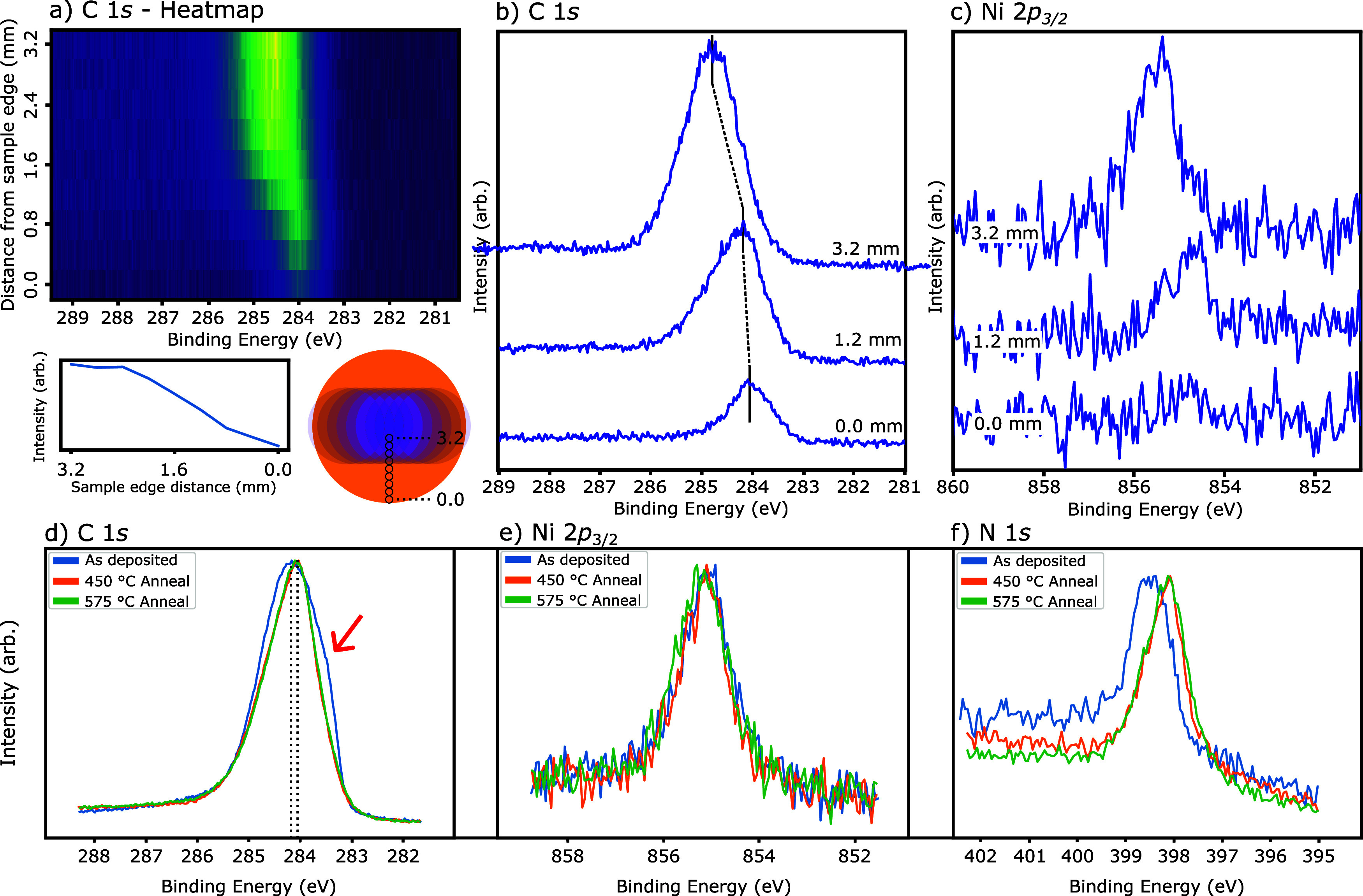
XPS characterization of **NiPP** on Au(111). (a) Normal incidence XPS, C 1*s* region, as a function of sample position. Coverage is seen to increase from edge to center of sample. Inset below shows the Au(111) surface, with the blue overlapping 2 mm deposition spot raster scanned across 10 positions on the surface (acquired after annealing at 575 °C). Black circles indicate the location of the beam incidence. XPS spectra for the (b) C 1*s* and (c) Ni 2*p*_3/2_ regions (acquired after annealing at 450 °C); the position of the C 1*s* peak shifts to lower BE in the lower coverage regions at the edge of the sample. (d–f) show XPS of the C 1*s*, Ni 2*p*_3/2_, and N 1*s* regions for as-deposited **NiPP** on Au(111) and following annealing to 450 and 575 °C (acquired at positions >2.0 mm from the sample center). Red arrow in (d) indicates a shoulder, attributed to contaminant material, which is removed following annealing.

To facilitate further chemical and structural analysis of the on-surface synthesis of porphyrin–graphene nanoribbons, synchrotron-based XPS, NEXAFS, and XSW measurements were performed. As the **NiPP**/Au(111) sample is prepared by ESD (see Experimental Section for details), a nonuniform molecular coverage is obtained. Figure 3a shows the variation in the XPS signal for the C 1*s* region as a function of sample position. The intensity of the C 1*s* signal increased from the edge to the middle of the sample. At the edge of the sample, the broad carbon peak is centered at ∼284.0 eV binding energy (BE) which shifts to higher BE, ∼284.6 eV, at the center of the sample (Figure 3b, acquired following annealing to 450 °C). The shift to a lower BE for lower coverage, at the sample edge, is assigned to increased screening via a molecule–substrate interaction. A similar shift is observed for the Ni 2*p*_3/2_ signal (Figure 3c), indicating that at the edge of the sample, the Ni atoms at the core of the porphyrin units are interacting with the substrate, while toward the center, some Ni species are interacting less strongly with the surface, which we assign to some sections of the polymer lying either atop contaminant material or regions of polymer.

In order to compare XPS information with acquired STM images, we consider the effect of annealing within the low coverage regions at the edge of the sample (acquired at positions >2.0 mm from the sample center where we expect a monolayer coverage). Figure 3d shows the evolution of the C 1*s* XPS signal as a function of annealing. Following annealing to 450 °C, a shoulder to the low BE side of the peak is removed (attributed to the removal of contaminant material) and results in a slight shift of the peak maxima to lower BE. We assign this shift, and change in peak shape, to an effect of the on-surface ring closing (as previously observed for tetraphenylporphyrin on Au(111)^20^) and formation of **GPNR**. The single nitrogen peak observed in the N 1*s* region (Figure 3f) is characteristic of metalloporphyrin species and suggests that Ni remains within the porphyrin macrocycle during annealing. The single Ni environment at 855.4 eV BE observed before and after annealing (Figure 3e) supports this assignment. In line with the carbon environment, following annealing, a shift in the BE of the N 1*s* peak is seen (∼398.5 to ∼398.0 eV BE), which is assigned to an increase in interaction between nitrogen atoms within the porphyrin core and the Au(111) substrate following the “flattening” of **NiPP** to form **PGNR**.

**Figure 4:**
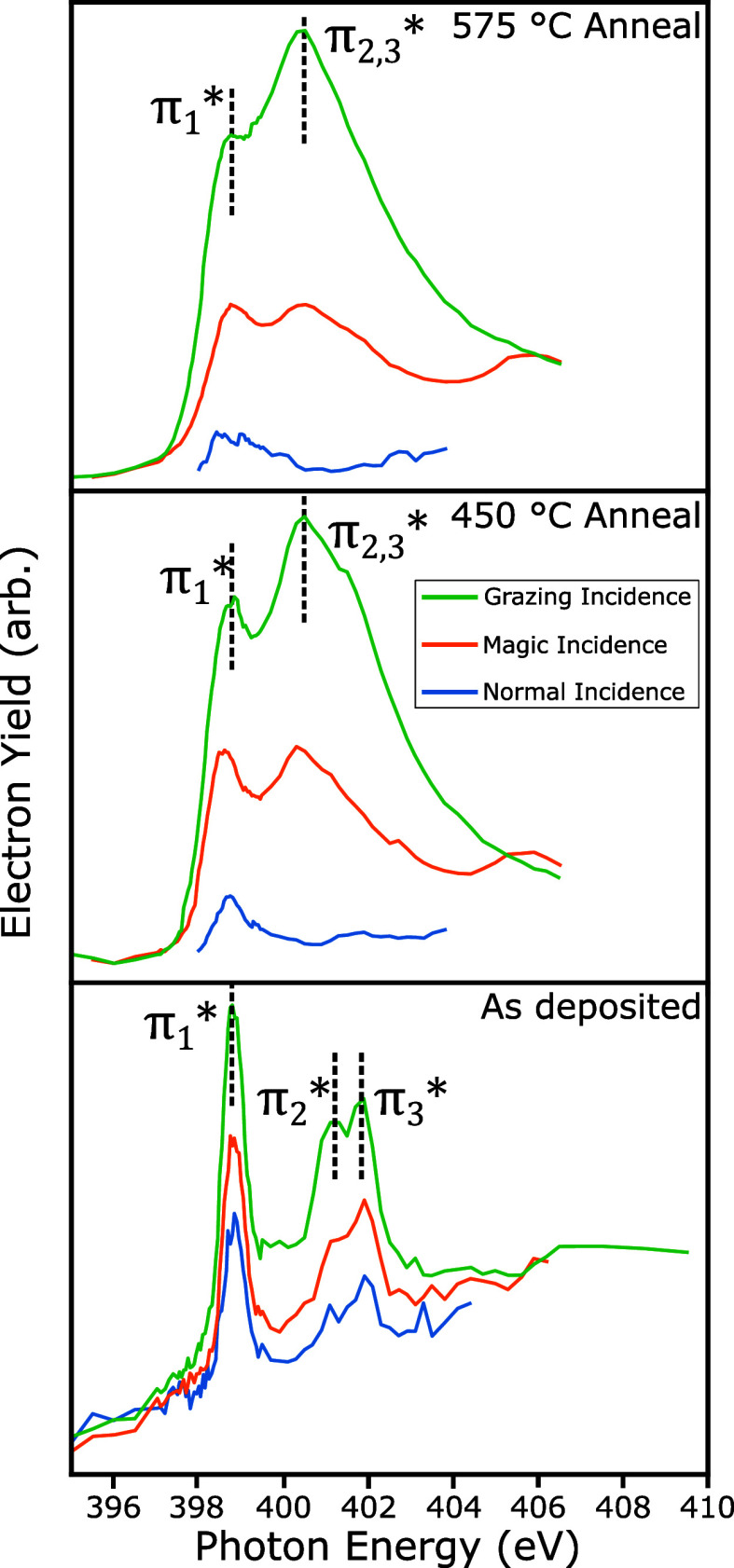
Angle-resolved NEXAFS spectra acquired at the nitrogen K-edge for **NiPP** on Au(111) as a function of annealing temperature: as deposited, 450 °C annealing, and 575 °C anneal. Resonances are labeled and discussed within main text.

Angle-resolved NEXAFS data acquired at the nitrogen K-edge provide information on the structural changes occurring within **NiPP** during the on-surface synthesis of **PGNR** and support the ring-closing and flattening reaction proposed based upon the STM and XPS data. In common with nitrogen K-edge NEXAFS for metalloporphyrins,^43−46^ resonances for as-deposited **NiPP** are observed at 398.8 eV (π_1_^*^), 401.2 eV (π_2_^*^), and 401.9 eV (π_3_^*^) (see Figure 4). Prior to annealing, the polymer shows limited dichroism and the reduction in intensity of the π_1_^*^, π_2_^*^, and π_3_^*^ peaks from grazing to normal incidence indicates an average tilt angle of ∼43° (which may correspond to a random ordering) for the core of the porphyrin relative to the plane of the surface (tilt angles obtained via the “ratio method” detailed in ref (47)). The π_1_^*^ resonance has previously been assigned to the LUMO (lowest unoccupied molecular orbital) for porphyrin species, and DFT studies indicate that this π-type orbital is located exclusively at the porphyrin core, including the nitrogen atoms.^44^ The angle of the porphyrin core to the substrate is in agreement with a model where the flexibility of the precursor allows the porphyrin units (containing nitrogen environments) to rotate around the C–C axis connecting them to the “graphitic precursor-unit” along the length of the polymer (see Figure 1c), similar to the canting of anthracene units within the intermediate reaction step of the on-surface synthesis of a graphene nanoribbon.^2^

Following each of the sequential annealing steps, the NEXAFS data reveal an increase in dichroism (Figure 4) corresponding to a reduction in the angle of the porphyrin core relative to the (111) surface plane. The π_2_^*^ and π_3_^*^ peaks are seen to broaden and merge (labeled as π_2, 3_^*^, 400.5 eV), while the position of the π_1_^*^ resonance is constant around 398.8 eV. After annealing to 450 °C, the average tilt angle is calculated, from the π_1_^*^ resonance, to be ∼28°, which further reduces to ∼22° following annealing to 575 °C. This confirms that the change in structure observed in STM is linked to flattening (and on-surface ring-closing reaction) where porphyrin species within **PGNR** are roughly parallel to the surface plane. Interestingly, the reduction in intensity of the π_1_^*^ resonance, relative to π_2_^*^/π_3_^*^ and π_2,3_^*^, following annealing, indicates an increased interaction with the substrate due to the partial filling of the π_1_^*^ molecular orbital.^45,48^ This reduction in intensity is therefore attributed to a flattening of the nanoribbon and a corresponding enhanced interaction between porphyrin core and metallic substrate (as indicated by the observed shift in BE within the XPS data).

Normal incidence X-ray standing wave (NIXSW) analysis was performed on **NiPP**/Au(111) using the (111) Bragg reflection (nominally 2630 eV). NIXSW is a chemically sensitive technique which allows the structure and adsorption positions of specific chemical elements in specific environments to be addressed.^49^

Within NIXSW, we consider the vertical adsorption of atomic species within specific chemical environments. Here, we obtain experimental values of *C*_f_ and *C*_p_ for the nitrogen species within the core of the porphyrin (from the single chemical environment identified within N 1*s* XPS data) and values for the ensemble of carbon environments; these values are shown in Table 1. Information on the degree of order, coherent fraction (*C*_f_), and the “average height”, coherent position (*C*_p_), of the specific chemical species relative to the projection of the bulk planes (i.e., in the case where no reconstruction, or relaxation, of the substrate occurs, *C*_p_ would define the height above the topmost surface layer) are obtained. Conceptually, *C*_f_ ≈ 1 would indicate a single adsorption site for the atomic species probed, while *C*_f_ ≈ 0 suggests adsorption at multiple sites or random ordering. The coherent position can generally be considered in relation to a mean adsorption height (*d*_*h*,*k*,*l*_) expressed as a function of the separation of the (*h*,*k*,*l*) planes, *D*_*h*,*k*,*l*_, such that *d*_*h*,*k*,*l*_ = (*n* + *C*_p_)*D*_*h*,*k*,*l*_, where *n* is an integer. The as-deposited **NiPP** material exhibits a low value of *C*_f_ for carbon species (0.08 ± 0.02), which implies no vertical ordering relative to the surface, i.e., a disordered polymer (NB as the value of *C*_f_ is effectively zero, no inference can be made from the value of *C*_p_). Following sequential annealing to 450 and 575 °C, an increase in the value of *C*_f_ is observed (0.19 ± 0.02 to 0.25 ± 0.02), indicating that there is an increase in order but that the carbon species present occupy multiple heights (again, the low coherent fraction means that it is not possible to meaningfully interpret the coherent position). The increase in *C*_f_ is in agreement with the expected outcome of forming graphitic material (i.e., an overall flattening of the polymers). In agreement with the STM data presented in Figures 1 and 2, the relatively low value of *C*_f_ indicates incomplete ring-closing within the polymer, which is expected to result in a range of carbon adsorption heights.

**Table 1:** Values for Coherent Fraction (*C*_f_) and Coherent Position (*C*_*p*_) Obtained from NIXSW Analysis for **NiPP** on Au(111) for the As-Deposited Material and Following Sequential Annealing to 450 and 575 °C to Form **PGNR**

**NiPP**	element	*C*_f_	*C*_p_
as-deposited	carbon	0.08 ± 0.02	0.01 ± 0.03
450 °C anneal	carbon	0.19 ± 0.02	0.73 ± 0.03
450 °C anneal	nitrogen	0.2 ± 0.1	0.3 ± 0.1
575 °C anneal	carbon	0.25 ± 0.02	0.45 ± 0.02
575 °C anneal	nitrogen	0.6 ± 0.1	0.36 ± 0.07

Analysis of the nitrogen species by NIXSW reveals that a significant level of order (*C*_f_ = 0.6 ± 0.1) is achieved following annealing to 575 °C. This increase in order following annealing (compare with *C*_f_ = 0.2 ± 0.1 prior to annealing) supports the interpretation that the on-surface reaction results in flattening of the porphyrin–graphene nanoribbons. Although this value of *C*_f_ does not indicate a high degree of order, it is comparable with studies of tetraphenylporphyrin (TPP) on Au(111)^21^ and zinc-porphine (ZnP) on Ag(111)^50^ where quasi-2D structures are observed and as such supports the formation of **PGNR**.

## Conclusions

We have demonstrated that porphyrin-doped graphene nanoribbons can be formed via a combination of atomically precise solution-based synthesis and on-surface reaction pathways. The conversion of a Ni-porphyrin polymer to a porphyrin–graphene nanoribbon has been characterized by STM, XPS, NEXAFS, and NIXSW providing confirmation that (i) the conversion of the **NiPP** polymer precursor to **PGNR** proceeds via a ring-closing reaction resulting in the “flattening” of the **PGNR** and (ii) the nitrogen and nickel chemical environments within the porphyrin core, as well as the morphology of the porphyrin macrocycle, remain unchanged during the formation of **PGNR,** indicating that Ni-porphyrin units are incorporated into the nanoribbon.

## Experimental Section

### Materials and Sample Preparation

The **NiPP** used in this study was synthesized and characterized, as reported previously.^24^ Samples of the Ni-porphyrin polymer material (**NiPP)** (Figure 1a), supported on Au(111) substrates, were prepared under vacuum conditions via electrospray deposition (see description in ref (39)). Au(111) substrates were prepared in UHV, prior to molecular deposition, by cycles of Ar^+^ sputtering (1 kV) and annealing (500 °C). The resultant clean gold surface was characterized by XPS (with a featureless C 1*s* region observed). The ESD source used was manufactured by Molecularspray Ltd. and mounted on a SPECS DeviSim NAP-XPS system at the University of Nottingham. **NiPP**(24) was dissolved in a mixture of toluene:methanol (3:1 ratio) to produce a solution containing 100 μg per mL. Electrospray ionization was initiated using a potential of 1.2 kV. Samples were prepared using (i) a “single-spot” deposition, with the molecular beam incident upon a single site on the sample for 40 min (spot diameter of ∼2 mm), or (ii) a “raster” deposition, where a series of 10 depositions, each 5 min in duration, were performed along the horizontal axis of the sample with 0.5 mm between each deposition site. The second approach allowed uniform coverage to be obtained over a large area of the sample.

### Scanning Tunneling Microscopy (STM)

Scanning tunneling microscopy (STM) experiments were performed using a Scienta Omicron POLAR low-temperature STM system operating under ultrahigh vacuum (UHV) conditions with a base pressure of better than 3 × 10^–10^ mbar. All samples were prepared by the “single spot” ESD procedure described above and transferred to the UHV-STM system using a vacuum suitcase (NextGeneration UHV Suitcase, Ferrovac AG). The STM was cooled to liquid helium temperatures, with a sample temperature of 4.7 K. All STM measurements were performed in constant current mode using electrochemically etched tungsten tips optimized by controlled indentation into the Au(111) single-crystal substrate [bias applied relative to the sample]. Differential conductance maps were measured in constant current mode and were generated using a lock-in amplifier output that applied a 20 mV signal at 2153 Hz in addition to the sample bias.

### XPS, NEXAFS, and NIXSW

Synchrotron-based XPS as well as NEXAFS and NIXSW measurements were performed at the I09 beamline^51^ at Diamond Light Source. **NiPP**/Au(111) samples were prepared in Nottingham (as described above) and transferred to IO9 via a Vacuum Suitcase (NextGeneration UHV Suitcase, Ferrovac AG). The I09 beamline utilizes two undulator sources allowing access to “soft” X-rays (100–2000 eV) and “hard” X-rays (2100–18000 eV). The “hard” X-rays were monochromated by a Si double-crystal monochromator, and the “soft” X-ray beam was monochromated by a plane grating monochromator. The XP spectra were acquired using a VG Scienta EW4000 HAXPES analyzer mounted perpendicular to the incoming light (light is linearly polarized in the horizontal plane). The binding energies were defined relative to the Fermi level of the substrate. Reflectivity curves were obtained from a fluorescent plate mounted in the port through which the synchrotron light is incident. The curves were then acquired by using a CCD camera mounted on a window opposite to the port. Reflectivity curves were fitted to determine the phase of the X-ray standing wave in addition to modeling peak broadening due to experimental uncertainties. Nondipolar effects in the photoelectron yield were modeled using a backward–forward asymmetry parameter, Q, derived from the calculations of Nefedov et al.^52,53^ Due to the large acceptance angle of the EW4000 analyzer (±30°), an effective emission angle of 15° was used for the (111) reflection, with respect to the surface plane. The hard X-ray had a beam spot size of 0.02 mm^2^, and the soft X-ray beam spot size was 0.16 mm^2^.

N 1*s*, C 1*s,* and Ni 2*p* core level XP spectra were obtained using photon energies of 550, 450, and 1160 eV, respectively. NEXAFS measurements were acquired at the nitrogen K-edge using photon energies in the range of 395–420 eV, while the photoelectron yield was detected via the hemispherical analyzer. Angular measurements were acquired at angles of 0° (normal incidence), 85° (grazing incidence), and 55° (“magic angle”). NIXSW measurements were performed using the (111) Bragg plane of the crystal (“hard” X-ray undulator on I09, with a nominal Bragg energy of 2630 eV). Each NIXSW measurement was repeated multiple times (10 times for the C 1*s* region and >8 times for the Ni 2*p*), with each new measurement performed at a different sample location to avoid beam damage. Before and after each XSW measurement, core level spectra for C 1*s* (*h*ν = 2620 eV) were obtained in order to monitor possible beam damage, with no significant changes observed. The sample was cooled using liquid nitrogen to reduce the effects of beam damage during data acquisition. A reflectivity curve was measured prior to each X-ray standing wave measurement to check the quality of the new areas of the surface and ensure that the energy range of each spectrum is the same with respect to the Bragg energy. The XP spectra acquired during the XSW measurement are fit with a combination of Gaussian and Doniach–Šunjić line shapes^54^; a single chemical environment is fit for both carbon and nitrogen species. The sample was characterized in the as-deposited state and following annealing to 450 and 575 °C in order to assess the outcome of the on-surface reaction.

## Supporting Information Available

Details of electrospray deposition, additional STM and *dI*/*dV* characterization, data supporting the NEXAFS and XSW analysis, and experimental data on which this work is based, may be found at 10.17639/nott.7415 (PDF)
