# Supplementary material for: On-Surface Synthesis of Ni-Porphyrin-Doped Graphene Nanoribbons
Source: ACS Nano. 2024 Nov 25;18(49):33390–7. doi: 10.1021/acsnano.4c09188 (PMC11636262; doi:10.1021/acsnano.4c09188)
Supplement: Supplementary file 1 — nn4c09188_si_001.pdf [file nn4c09188_si_001.pdf]

On-surface synthesis of Ni-porphyrin doped graphene nanoribbons

Matthew Edmondson,[1] Michale Clarke, [1] James N. O'Shea, [1] Qiang Chen,[2] Harry L. Anderson, [2] and Alex Saywell[1*]

[1] School of Physics & Astronomy, University of Nottingham, Nottingham, NG7 2RD, UK.

[2] Department of Chemistry, University of Oxford, Chemistry Research Laboratory, Oxford OX1 3TA, UK.

* Corresponding author A.S. (alex.saywell@nottingham.ac.uk)

Supporting Information

Contents

Additional Experimental Details S2

STM analysis of NiPP S2

Electrospray deposition (ESD) S3

dI/dV maps of PGNR S3

NEXAFS S4

NIXSW S4

Additional Experimental Details

STM analysis of NiPP

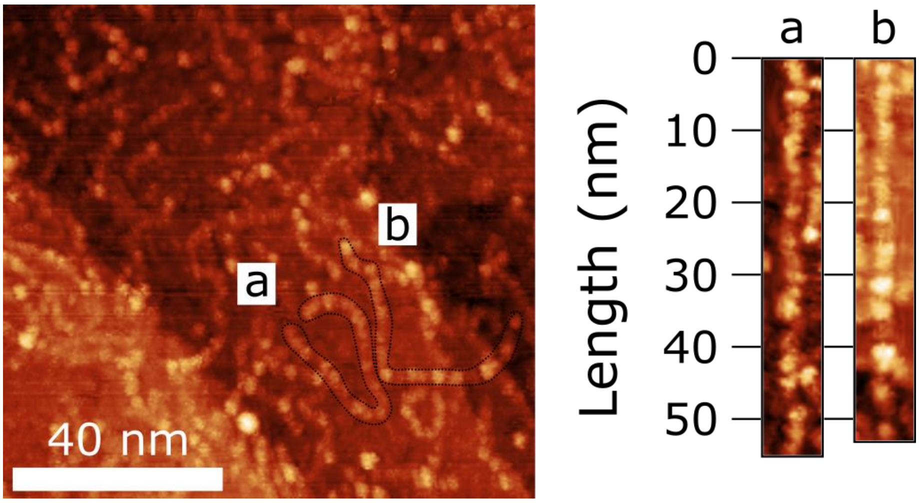

*Figure S1: STM topographic image showing the as-deposited **NiPP** material on Au(111). Two segments from the **NiPP** polymer chains,* a *and* b*, are outlined. Topographic details along the length of these segments are presented on the right of the figure. Due to crossing points between polymers, it is non-trivial to determine the total length of any given chain. The maximum continuous chain length measured is ~ 55nm. Image parameters: Image acquired at 78 K, -1.5 V and 50 pA.*

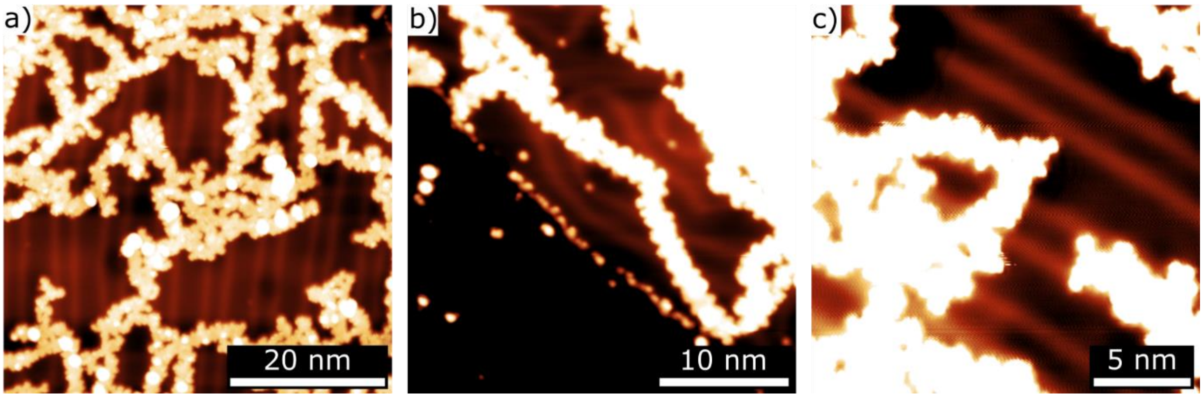

Figure S2: STM topographs acquired following annealing to 475°C with enhanced contrast to provide visibility of the Au(111) surface reconstruction. No obvious interaction between nanoribbon and the surface, that disrupts the reconstruction, is observed. Image parameters: All images acquired at 4.7 K, (a) -2.0 V and 50 pA, (b) -2.0V and 100 pA, and (c) -0.2 V and 50 pA.

Electrospray deposition (ESD)

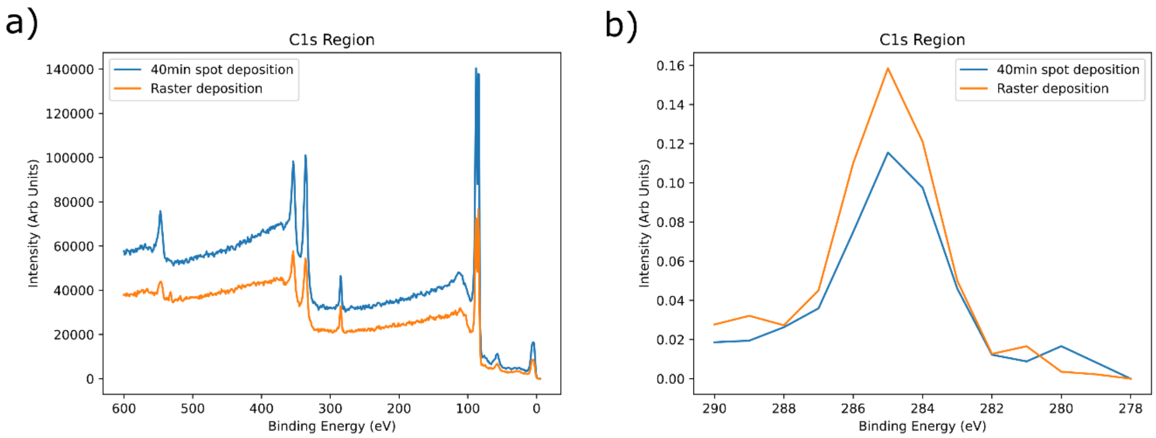

Figure S3: Coverage calibration of ***NiPP*** *following electrospray deposition onto Au(111). (a) X-ray photoelectron spectroscopy (XPS) 'wide scans' acquired for the 40 min spot deposition sample (from which the STM images were acquired) and the raster deposition sample (from which the XPS, NEXAFS and XSW were acquired). (b) C1s* XPS *region from the 'wide scans', normalised to the Au4f peaks: coverages are comparable and assigned to < 1 ML, based upon STM data.*

dI/dV maps of PGNR

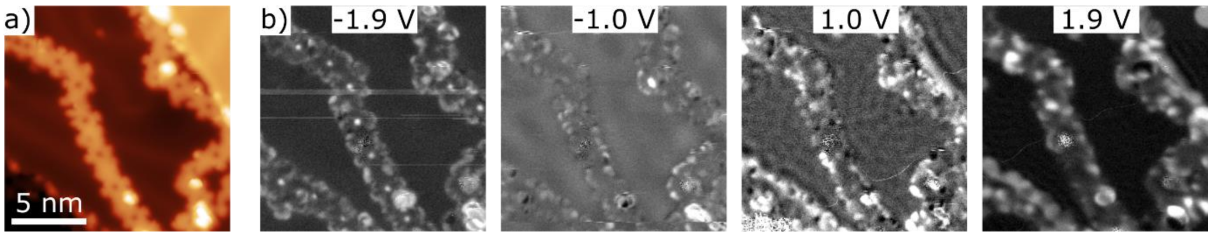

Figure S4: Examples of dI/dV (differential conductance) maps acquire for the on-surface synthesised ***PGNR*** *material for sample biases from +2 V to -2 V. (a) Topography image of ring-closed Nanoribbon. (b) dI/dV maps of the same area taken with a bias of -1.9 V, -1.0 V, 1.0 V and 1.9 V. The topography image and all dI/dV maps were taken at 100 pA in constant current mode. The features in the nanoribbon that are assigned to the Ni core of the porphyrin are visible as bright spots at biases from -2.0 V to -1.2 V. At other bias values such features are not visible.*

NEXAFS

Table S1: Binding energies and bond angles of π resonances obtained from nitrogen K-edge NEXAFS (see Fig.4 in the manuscript). Bond angles are measured relative to the surface plane, therefore small bond angles indicate that the plane of the porphyrin core is approximately parallel with the substrate [citation: J. Stöhr and D. A. Outka, Phys. Rev. B, 1987, 36, 7891–7905].*

Sample Temp (°C)	Properties	π*1	π*2	π*3
RT	*Photon Energy (eV)*	398.8	401.1	401.9
RT	*Angle (°)*	43.1	41.4	43.2
		**π*1**	**π*2-3**	
450	*Photon Energy (eV)*	398.6	400.4	
450	*Angle (°)*	28	14.9	
575	*Photon Energy (eV)*	398.8	400.4	
575	*Angle (°)*	22.2	9.2	

NIXSW

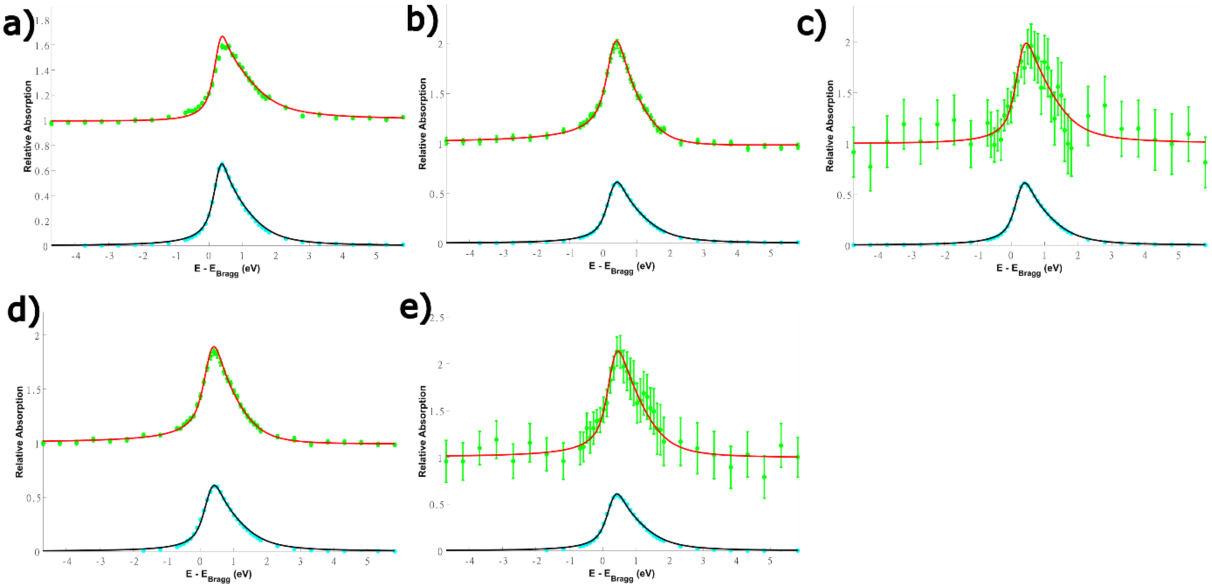

Figure S5: Details of the XSW adsorption profiles for the datasets displayed within Table 1 of the manuscript. XSW adsorption data and fit for: (a) the C1s region pre-anneal, (b) the C1s region post 450°C anneal, (c) the N1s region post 450°C anneal, (d) the C1s region post 575°C anneal, and (e) the N1s region post 575°C anneal.
